# Salvaged single-unit cord blood transplantation for 26 patients with hematologic malignancies not in remission

**DOI:** 10.1590/1414-431X20154389

**Published:** 2015-03-27

**Authors:** W. Yao, C.C. Zheng, H.L. Liu, L.Q. Geng, B.L. Tang, J. Tong, X.Y. Zhu, K.D. Song, P. Qiang, Z.M. Sun

**Affiliations:** 1 Shandong University, School of Medicine, Jinan, China; 2 Department of Hematology, Anhui Provincial Hospital, Anhui Medical University, Hefei, China

**Keywords:** Umbilical cord blood transplantation, Hematologic malignancies, Non-remission

## Abstract

Treatments for patients with hematologic malignancies not in remission are limited, but a few clinical studies have investigated the effects of salvaged unrelated cord blood transplantation (CBT). We retrospectively studied 19 patients with acute leukemia, 5 with myelodysplastic syndrome (MDS with refractory anemia with excess blasts [RAEB]), and 2 with non-Hodgkin's lymphoma who received 1 CBT unit ≤2 loci human leukocyte antigen (HLA)-mismatched after undergoing myeloablative conditioning regimens between July 2005 and July 2014. All of them were in non-remission before transplantation. The infused total nucleated cell (TNC) dose was 4.07 (range 2.76-6.02)×10^7^/kg and that of CD34^+^ stem cells was 2.08 (range 0.99-8.65)×10^5^/kg. All patients were engrafted with neutrophils that exceeded 0.5×10^9^/L on median day +17 (range 14-37 days) and had platelet counts of >20×10^9^/L on median day +35 (range 17-70 days). Sixteen patients (61.5%) experienced pre-engraftment syndrome (PES), and six (23.1%) patients progressed to acute graft-versus-host disease (GVHD). The cumulative incidence rates of II-IV acute GVHD and chronic GVHD were 50% and 26.9%, respectively. After a median follow-up of 27 months (range 5-74), 14 patients survived and 3 relapsed. The estimated 2-year overall survival (OS), disease-free survival (DFS), and non-relapse mortality (NRM) rates were 50.5%, 40.3%, and 35.2%, respectively. Salvaged CBT might be a promising modality for treating hematologic malignancies, even in patients with a high leukemia burden.

## Introduction

Patients with hematologic malignancies not in remission have very poor outcomes, and allogeneic hematopoietic stem cell transplantation (allo-HSCT) is the only possibly effective treatment. However, only 25% of patients have a human-leukocyte antigen (HLA)-identical sibling. In China, the chance of having an HLA-identical sibling is very low owing to the one-child policy. Haploidentical transplantation is dependent on donor availability and post-transplantation adoptive cellular immunotherapy, but it may be complicated by a high risk of graft failure and relapse (1). Umbilical cord blood transplantation (CBT) has the advantages of being rapidly available, and having a lower incidence of graft-versus-host disease (GVHD) and less strict HLA-matching requirements owing to lower numbers and more immature T lymphocytes. In addition, CBT procedures can safely provide a strong graft versus leukemia/lymphoma (GvL) effect (2,3), especially for high-risk hematologic malignancies, and a high disease-free survival rate (4). For adults, registry-based studies have established CBT as a safe and feasible alternative to bone marrow transplantation (BMT) when a matched sibling donor is not available (5,6). However, information on disease-specific outcomes is limited, in particular for patients who are not in remission before CBT. This report describes 26 patients with myeloid and lymphoid malignancies who were not in remission at the time of transplantation and who were treated with myeloablative regimens in preparation for CBT at our center. They were followed for a median of 27 months from the date of transplantation.

## Patients and Methods

### Patients

Twenty-six consecutive patients underwent allogeneic transplantation in our center using one unrelated cord blood unit between July 2005 and July 2014. Their demographic and clinical details are shown in Tables 1 and 2. All patients had been diagnosed with hematologic malignancies. They were not in remission, defined as more than 5% blasts in bone marrow (BM). They all lacked a 5/6 or fully HLA-matched related or unrelated donor. Their median age was 13 years (range 6-32 years), and their median weight before transplantation was 45 kg (range 18-73 kg).

**Table:**
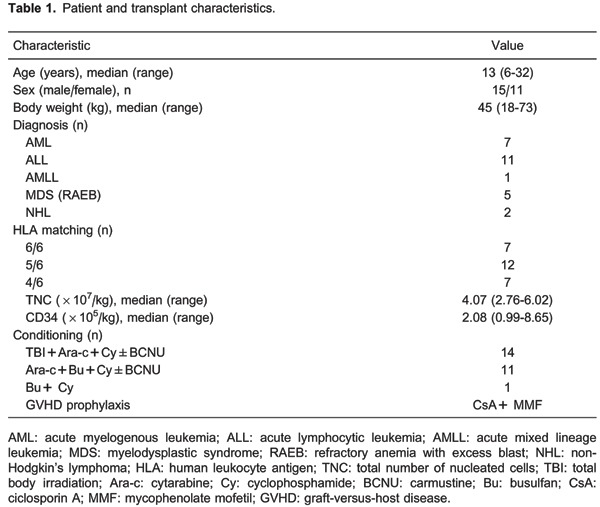

**Table:**
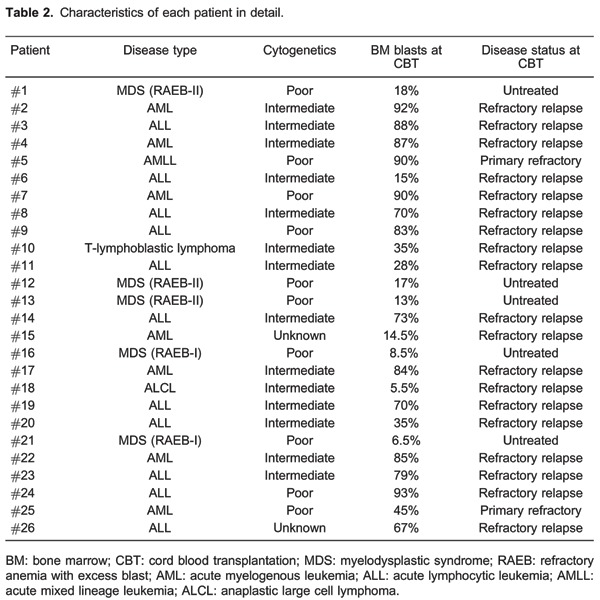

### Transplantation procedures

All patients underwent a myeloablative pre-transplantation conditioning regimen and 1 CBT unit from public CB banks in China. The myeloablative conditioning regimens were classified into 3 groups: 1) total body irradiation (TBI)-based regimens (TBI of 3 Gy twice daily on days -7 and -6, cytosine arabinoside [Ara-C] 2 g/m^2^ twice daily on days -5 and -4, and cyclophosphamide [Cy] 60 mg/kg once daily on days -3 and -2 ± carmustine [BCNU] 250 mg/m^2^ on day -5); 2) Ara-c 2 g/m^2^ twice daily on days -9 and -8 and busulfan (Bu)+Cy±BCNU at the same dosages as the previous regimen; or 3) Bu 4 mg/kg once daily on days -7 to -4, and Cy 60 mg/kg once daily on days -3 and -2. In patients with myeloid malignancies, granulocyte colony-stimulating factor (G-CSF) was infused before cytarabine. The GVHD prophylaxis consisted of intravenous ciclosporin (CsA; Novartis, Switzerland) and mycophenolate mofetil (MMF; Roche, Switzerland). CsA was administered intravenously at a dose of 3 mg/kg daily from day -1, and MMF was administered orally from day +1 at a dose of 25 or 30 mg/kg daily. G-CSF was used from day +6 to facilitate neutrophil engraftment until the white blood cell (WBC) count increased to at least 4×10^9^/L for 2 consecutive days. Infection prophylaxis consisted of oral itraconazole (Janssen, China) and aciclovir (GlaxoSmithKline, UK), followed by sulfamethoxazole (Baiyunshan, China) to prevent *Pneumocystis jirovecii* infection.

### Assessment of chimerism, engraftment, and GVHD

Confirmation of donor engraftment was made either by cytogenetics for sex-mismatched transplants or by chimerism analysis of flow-sorted peripheral blood T cells and nucleated marrow cells. Polymerase chain reaction (PCR) of highly polymorphic short tandem repeat (STR) units was performed. Complete donor chimerism was defined by the lack of a previously determined recipient-specific STR on polyacrylamide gel analysis. These assays could detect mixed chimerism if more than 5% donor or recipient cells were present. The day of myeloid engraftment was defined as the first of 3 consecutive days when the absolute neutrophil count exceeded 0.5×10^9^/L. The day of platelet engraftment was defined as the day when the absolute platelet count exceeded 20×10^9^/L without platelet transfusion. Both acute and chronic GVHD (aGVHD and cGVHD) were diagnosed and graded based on published criteria (7,8).

### Statistical analysis

The endpoints of this study were engraftment, aGVHD and cGVHD, infectious complications, non-relapse mortality (NRM), relapse rate, disease-free survival (DFS), and overall survival (OS). Survival rates were calculated by the Kaplan-Meier method with the log-rank test. The cumulative incidents rates of NRM, relapse, aGVHD, and cGVHD were computed to determine the presence of competing risks (9). P<0.05 was considered to indicate statistical significance in all analyses.

## Results

### Engraftment, transplantation toxicity, and infections

All patients were engrafted with neutrophils that exceeded 0.5×10^9^/L on median day +17 (range 14-37 days) and had platelet counts >20×10^9^/L on median day +35 (range 17-70 days). Three patients died before platelet engraftment after CBT because of severe GVHD and intestinal infection. By day 21 post CBT these patients had all converted to full donor chimerism (FDC). Sixteen (61.5%) patients experienced pre-engraftment syndrome (PES), and 6 (23.1%) patients progressed to aGVHD.

Severe pneumonia and intestinal infection occurred in six patients and eventually led to death. Sepsis was documented in five patients but was controlled by antibiotics during the agranulocytosis period after CBT. Seven patients developed cytomegalovirus (CMV) viremia (CMV-DNA >10^3^ copies/mL) but were negative after antiviral treatment. None developed CMV-associated diseases. Three patients experienced hemorrhagic cystitis and were cured by adequate hydration and urine alkalinization.

### GVHD

The cumulative incidence of aGVHD was 50% (95% confidence interval [CI]=31.0-69.0%), and grade II-IV aGVHD was diagnosed in 26.9% (95% CI=9.9-43.9%). Thirteen patients developed aGVHD. Three patients died of grade IV aGVHD before day +100. In the remaining 10 patients, aGVHD was under control after treatment with CsA or tacrolimus combined with methylprednisolone and MMF. With a median follow-up of 27 months (range 5-74 months) for surviving patients, the cumulative incidence of cGVHD was 26.9% (95% CI=9.9-43.9%), and there was no extensive cGVHD.

### Outcome

**Figure 1:**
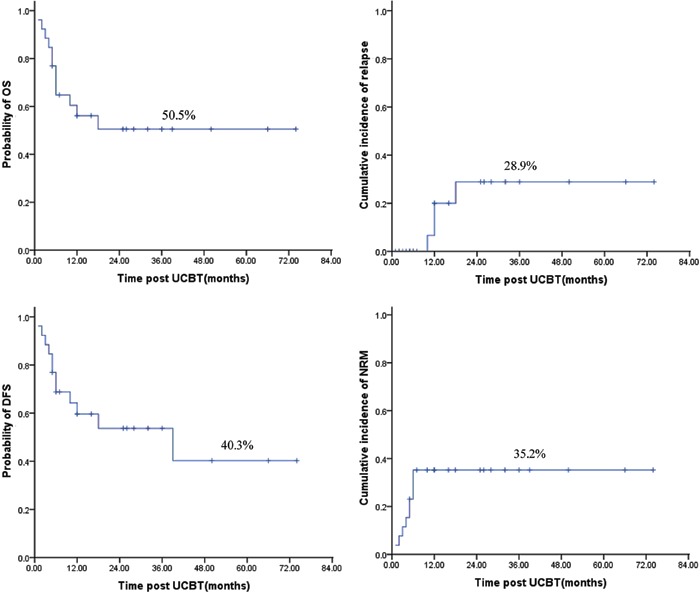
Overall survival (OS), disease-free survival (DFS), relapse and non-relapse mortality (NRM) in patients undergoing cord blood transplantation. UCBT: umbilical cord blood transplantation.

As of July 2014, the median follow-up was 27 months (range 5-74 months). In all, 14 patients survived who were in sustained complete remission (CR) and 12 died, 3 from relapse and 9 from NRM (6 from severe infection, 3 from aGVHD). The estimated 2-year OS, DFS, relapse, and NRM rates were 50.5% (95% CI=31.3-69.7%), 40.3% (95% CI=21.4-59.2%), 28.9% (95% CI=11.5-46.3%), and 35.2% (95%CI=16.8-53.6%), respectively (Figure 1).

## Discussion

Despite the important growth in CBT procedures worldwide, especially for adult patients with high-risk hematologic malignancies (4,10,11), our study is unique in that none of the patients enrolled were in remission at the time of transplantation. Although we assessed a high-risk cohort, the estimated 2-year OS and DFS were 50.5% and 40.3%, respectively, which are comparable with those of other studies (though only a subset of the patients enrolled were in advanced stages before CBT) (4,10,11).

Unrelated donor CBT is associated with a significant engraftment failure rate (10,12,13). This unfavorable outcome is due to a low cell dose infused and the frequent HLA disparity between the donor and recipient. In our study, as in those carried out at the University of Tokyo (14) and in Spain (11), hematopoietic recovery in CBT recipients was rapid. No primary or secondary graft failure was observed, and engraftments were achieved within 21 days in all engrafted patients. The intensification of conditioning by administering additional cytarabine in the setting of TBI-CY or BU-CY, which remain the most common myeloablative conditioning regimens for allogeneic HSCT without the use of an anti-T cell antibody, was sufficiently effective to eradicate malignant cells and achieve engraftment. In addition, we combined cytarabine with G-CSF infusion in patients with myeloid malignancies based on the hypothesis that G-CSF increases the susceptibility of myeloid leukemic cells to cytarabine (15,16).

Three risk factors for grade II-IV aGVHD after CBT were identified in multiple regression analysis: use of 2 CB units, use of non-myeloablative conditioning, and absence of antithymocyte globulin in the conditioning regimen (17). As was previously reported (4,11,17), we confirmed a relatively low incidence of aGVHD/cGVHD after CBT despite the absence of antithymocyte globulin. This may be related to the use of one CB unit or to intensified myeloablative conditioning. In addition, MMF may be more effective than MTX in GVHD prophylaxis.

Regarding NRM, we observed a higher rate (35.2%) than that reported by the University of Tokyo study (15%) (3). However, other studies reported cumulative NRM incidence rates of 30% to 60% (6,11,18-22). The most important reason was disease status at transplantation, which was associated with extensive prior treatment and poor tolerance to transplant-associated toxicities that likely increased transplantation related mortality (TRM). We also noted higher mortality due to infection. Future investigations should focus on more effective preventive and therapeutic measures to manage infectious complications and improve outcomes.

The relapse rate was low and similar to those previously reported for patients with high-risk hematologic malignancies in the setting of CBT (10); it was even superior to that of haploidentical (40%) (23) and related or unrelated peripheral stem cell transplantation (32%) (24), confirming the high antileukemic efficacy of the procedure. Hence, OS and DFS were mainly influenced by NRM and the associated risk factors.

In conclusion, single-unit CBT with myeloablative conditioning can be carried out with acceptable toxicity and TRM and increases long-term remission in patients with hematologic malignancies who were not previously in remission. The limitations of this study are that it was a retrospective analysis of a small number of patients with relatively short follow-up periods in the survivors. Additional large-scale studies with long-term follow-up are needed to better evaluate the efficacy of this procedure.
